# Assessment of Visual Acuity and Stereopsis in Older Adults: A Comparison Between a Screening Application and Clinical Standards—A Feasibility Study

**DOI:** 10.3390/medicina62030517

**Published:** 2026-03-11

**Authors:** Dorottya Wiegand, Eszter Mikó-Baráth, Ildikó Telkes, Balázs Patczai, Adrienne Csutak, Vanda Agnes Nemes

**Affiliations:** 1 Institute of Physiology, Medical School, University of Pécs, Szigeti Str. 12, H-7624 Pécs, Hungary; wiegand.dorottya@pte.hu (D.W.); ildiko.telkes@aok.pte.hu (I.T.); vanda.nemes@aok.pte.hu (V.A.N.); 2 Department of Ophthalmology, Clinical Centre, Medical School, University of Pécs, Rákóczi Str. 2, H-7623 Pécs, Hungary; csutak.adrienne@pte.hu; 3 Department of Traumatology and Hand Surgery, Clinical Centre, Medical School, University of Pécs, Ifjúság Str. 13, H-7624 Pécs, Hungary; patczai.balazs@pte.hu

**Keywords:** screening application, visual acuity, stereopsis, aging, vision screening

## Abstract

*Background and Objectives:* Visual impairment and reduced stereovision significantly impact the quality of life and increase fall risk in older adults. While standard clinical assessment of visual functions is essential in this population, its use is often limited by the need for specialized equipment and trained personnel. Tablet-based screening tools offer a practical alternative but require clinical validation. This study aimed to assess the agreement, reliability, and diagnostic performance of a tablet-based screening application (index methods) compared to established clinical reference methods for assessing visual acuity (VA) and stereovision (SV) in adults over 60 years. *Materials and Methods:* This prospective, non-blinded, cross-sectional, feasibility study included two cohorts: a test–retest group of 24 older adults assessed twice within 7 days, and a clinical cross-sectional group of 135 participants recruited from primary care practices. VA was measured using tablet-based Landolt C test and compared with an ETDRS-style chart, while stereovision was assessed using tablet-based static and dynamic random dot stereograms and compared with the TNO stereotest. Agreement and reliability were evaluated using Bland–Altman analysis, intraclass correlation coefficients (ICC), and receiver operating characteristic (ROC) curves. *Results:* The index VA method demonstrated good test–retest reliability (ICC = 0.79) with no significant difference between repeated measurements. In the clinical cross-sectional group, visual acuity measurements showed a small mean bias (0.022 logMAR) between the index and reference methods, which remained within clinically acceptable limits, particularly in the intermediate acuity range. For stereovision, the index SV tests showed high test–retest agreement. Using a TNO cutoff of 480 arcsec, the index SV method demonstrated good diagnostic accuracy (AUC 0.87 for static and 0.85 for dynamic stimuli) with high sensitivity for detecting impaired stereovision. *Conclusions:* The tablet-based index method provided reliable and clinically comparable results for VA and SV assessments in older adults, supporting its potential use as a screening tool in primary care and community-based settings.

## 1. Introduction

Good eyesight is essential for independence and daily activities, even in old age. Visual impairment reduces quality of life, shortens life expectancy, increases the need for care, and can lead to social isolation, physical inactivity, and depression [1,2,3]. It also contributes to postural instability, raising the risk of fall-related fractures, morbidity, and mortality [4,5,6,7]. Therefore, systematic screening in the aging population is important, as gradual vision loss often goes unnoticed. The decay in visual functions can be quantified by visual acuity (VA) alone, while contrast sensitivity, visual fields, or stereovision (SV) offer valuable additional information [4,8].

A clinically accepted and validated method for measuring VA is the Early Treatment Diabetic Retinopathy Study (ETDRS) chart, providing precise and reliable readings in a clinical setting [9]. However, its use requires a trained examiner, specific environmental and technical conditions, which may be challenging to implement when resources are limited. Moreover, elderly or immobile patients may find it difficult to travel or visit another department for a vision test that could be done at the bedside.

Screening for impaired stereopsis in older adults is also essential, as reduced depth perception adversely affects daily activities such as driving or walking on uneven surfaces, significantly increasing the risk of injuries [1,10,11]. Although less commonly assessed, stereopsis is gaining increasing attention in this population [5,12]. In a previous study involving post-hip-fracture patients, we found that visual impairment, as defined by reduced stereopsis and monocular visual acuity, was significantly associated with increased fall risk, with an odds ratio (OR) of 4.88 (95% CI = 1.87–12.76, *p* = 0.0012) [13]. For comparison, reduced monocular visual acuity alone yielded an OR of 3.82 (95% CI = 1.50–9.73, *p* = 0.0049, unpublished data). These findings are consistent with earlier reports suggesting that reduced or absent stereopsis significantly increases fall risk in older adults [5,12].

Recent advances in digital platforms now allow the assessment of visual functions such as acuity, stereopsis, contrast sensitivity, color vision, and visual fields [14]. These tools offer several advantages over traditional methods, allowing testing in diverse settings—including bedside, outpatient, and remote environments like schools or rural areas—making them accessible to clinicians, researchers, and the public [15,16,17,18]. These applications often incorporate features of established clinical methods (e.g., controlled illumination, adjustable optotype size, randomization) and integrate adaptive threshold algorithms as well as cloud-based storage. To ensure their suitability for clinical use and large-scale screening programs, these digital tools must first be validated against established clinical standards [19]. Several studies show these apps can match or exceed traditional tests in performance [14,15,16,17,18].

In this study, the EuvisionTab^®^ digital platform, developed by the University of Pécs, Hungary, was used as the index method (hereinafter referred to as the index method) [20,21] and its performance was compared with established clinical methods (reference methods) for VA and stereopsis.

Specifically, the present study had three main objectives:

1. To compare VA and SV measurements with the index and the clinical reference methods, namely the ETDRS-style Landolt C chart for VA and the TNO stereotest for SV, to assess how accurately the index method identifies visual impairment in individuals over 60 years of age.
2. To examine the test–retest reliability of the index method for VA and SV in an elderly population.
3. To evaluate the diagnostic performance of the index stereotests and define optimal cutoffs for detecting impaired stereopsis in an aging population.

If sufficient agreement is demonstrated, this would support the feasibility of using mobile, tablet-based screening as a practical alternative to specialist-led examinations in primary care settings.

## 2. Materials and Methods

### 2.1. Participants

This prospective feasibility study included older adults who volunteered to participate. The study followed a non-blinded, cross-sectional design and involved two complementary groups with different study aims (Table 1). The first group (test–retest group) was examined under laboratory conditions at the Department of Physiology, University of Pécs. It included 24 participants (mean age: 65.96 years, SD = ±5.94, female: 45.8%) in good general health, with no known major eye diseases and relatively good vision with their usual glasses. Participants in this group completed the same visual tests twice, with a 4–7-day interval between sessions, to assess the repeatability of the measurements. 

The second group (Clinical cross-sectional group) consisted of 135 patients (mean age: 72.73 years, SD = ±6.95; female: 70%), recruited from two general practitioner offices in Baranya County, Hungary. In his more diverse group, most individuals had no, or stable, well-controlled chronic medical conditions. Participants were assessed once during a single session.

The study was approved by the Regional Research Ethics Committee of the University of Pécs (approval numbers 8363-PTE 2020 and 2023) and was conducted in accordance with institutional and national regulations, as well as the Declaration of Helsinki. All participants received detailed information about the study and provided written informed consent prior participation.

### 2.2. Testing Procedures

All participants were tested by the same two experienced examiners using the same equipment and identical conditions in a single session lasting approximately 30 min. Monocular and binocular visual acuities (VA) were measured while patients wore their distance correction, and reading glasses were used for stereovision (SV) when required. An overview of the tests is provided in Table 2.

The EuvisionTab^®^ software (version: 0.32), developed at the University of Pécs, Hungary, was used as the index method [13,20,21]. It enables flexible assessment of visual acuity (VA) and stereovision (SV) through adjustable stimulus parameters and standardized testing principles. The stimulus presentation and response registration were conducted on a 10.1-inch Samsung Galaxy Tab A tablet. Key technical parameters of the system are summarized in Box 1. to support reproducibility.

Box 1 **Technical details of the index method.**

   The EuvisionTab^®^ system is a tablet-based, examiner-guided digital platform for the assessment of visual acuity (VA) and stereovision (SV) [13,20,21]. The application runs on Android-based mobile devices and enables automated stimulus control and electronic data capture with secure cloud-based storage.

   **Visual acuity (VA) module**

   The VA test presents single Landolt C or Snellen E optotypes at a chosen examination distance (3–5 m). Stimulus size is modified adaptively according to participant responses using the Best Parameter Estimation by Sequential Testing (Best PEST) algorithm [22]. The system allows semi-continuous estimation (59 different optotype sizes) across a wide acuity range (−0.44–+1.3 logMAR) using logarithmic size scaling. Measurement parameters are aligned with ISO recommendations, where applicable [23,24].

   **Stereovision (SV) module**

   The SV module generates static and dynamic random-dot stereograms (SRDS and DRDS) using red–green anaglyph (R26 red and YG09 green gelatin filters) separation. Disparity-defined optotypes are presented binocularly at reading distance, while monocular control stimuli are used to confirm task understanding. Key stimulus parameters, including dot size and density, disparity magnitude, and refresh rate, are adjustable to control task difficulty and sensitivity.

### 2.3. Visual Acuity Tests

Visual acuity (VA) was assessed using both a clinical reference method and the tablet-based index method.

The reference method employed an ETDRS-style Landolt C chart built into a 23.6-inch LCD vision chart (VISIONIX VX22, Luneau Technology, Pont-de-l’Arche, France), following the *ISO 8596:2017* and *ISO 10938:2016* standards [23,24]. The display showed five high-contrast Landolt C optotypes per line, each in one of eight orientations. The correct identification at each line was followed by a reduction in optotype size. The VA threshold was defined as at least three correct identifications out of five per line [9].

In the test–retest group, the full VA range, including hyperacuity (logMAR < 0), was explored. In contrast, in the clinical cross-sectional group, measurements were terminated at 0 logMAR, the threshold for normal vision, to reflect typical clinical practice, without extension into the hyperacuity range. 

In case of the tablet-based index method, a total of 16 single Landolt C optotypes were presented sequentially, with randomly oriented gaps in one of eight directions. Optotype size was adaptively modified based on participant responses, using the Best PEST algorithm [22,25]. Participants indicated the gap orientation (e.g., top right), verbally or by pointing, and the examiner entered the responses manually on the tablet.

### 2.4. Stereovision Tests

Stereovision was similarly assessed using both a reference and the index method.

The reference was the paper-based TNO stereotest (Lameris OotechB.V., Utrecht, The Netherlands). Plate I (butterfly, 1980 arcsec,”) assessed the presence of coarse stereopsis, while Plates V and VI tested stereoacuity thresholds ranging from 480″ to 60″. Failure to detect the butterfly indicated absent (‘nil’) stereopsis.

The SV module of the index method presented both SRDS and DRDS, each featuring a disparity-defined Snellen E optotype in one of four random orientations. (Table 2). Stimulus disparity was fixed at 840″ for both RDS types, dot density was set at 8% for static and 0.7% for dynamic stimuli, with dynamic images refreshed at a rate of 30 Hz. Each test included 10 identical SRDS and DRDS presentations. Participants identified the orientation of the “E” either verbally or by tapping the screen. Control (monocularly visible) images were presented at the beginning to verify task comprehension. 

### 2.5. Statistical Analysis

Statistical analyses were performed using MedCalc for Windows, version 20.211 (MedCalc Software Ltd., Ostend, Belgium). For the test–retest group, Bland–Altman analysis and intraclass correlation were used to assess intra-individual VA differences. SV data were examined in crosstabs. Agreement between TNO-based classification and SRDS/DRDS outcomes was quantified using Cohen’s kappa statistics and overall percentage agreement.

The following steps were taken to analyze the VA and SV data from the clinical cross-sectional group:

1. VA outcomes: Cross-sectional descriptive outcomes are reported for both methods. Agreement between the tablet-based index test and the reference was evaluated with Bland–Altman method by calculating mean difference (bias) and 95% limits of agree-ment (LoA). A paired *t*-test was used to examine whether a systematic difference existed between the two methods. For comparability, VA values better than 0 logMAR obtained with the index method were truncated at 0, and the analysis focused on acuity values worse than 0 logMAR. This range included 130 of 189 measurements, and reflects the age and typical visual status of the study population.
2. SV outcomes: For the primary analysis, a TNO disparity level of 480″ was selected as the reference threshold, as it corresponds to the upper limit of stereoacuity typically reported in older adults [24,25,26] and showed the best agreement with index test results in our da-taset (see Appendix A). For the index stereotests (SRDS and DRDS), performance was evaluated using a pass–fail criterion defined as correctly identifying at least 5 out of 10 stimuli. This cutoff was selected based on a Bernoulli probability model (*p* < 0.01), as the probability of achieving ≥5 correct responses by random guessing in a 10-trial format is below 1%.Diagnostic performance of the index tests relative to the TNO-based classification was evaluated using Receiver Operating Characteristic (ROC) analysis, with pass–fail cutoffs optimized for sensitivity. The area under the ROC curve (AUC) and corresponding confidence intervals were calculated.

Additionally, McNemar’s test was applied to compare the proportion of participants classified as having impaired stereovision by the TNO test versus the index tests.

## 3. Results

The results are presented separately for the two study groups. No data was lost due to technical issues, though some patients could not complete all tests due to fatigue. No participants were excluded from the test-retest group, and altogether four individuals with very low monocular visual acuity were removed from the clinical cross-sectional group, resulting in a final sample of 131 participants. The descriptive results can be found in Table 1, while number of participants for each test is specified in Table 2. 

### 3.1. Test–Retest Group

#### 3.1.1. Visual Acuity

Test-retest reliability was evaluated for both methods (Table 3A). The tablet-based index method demonstrated good agreement between repeated measurements with no significant systematic difference between sessions and high reliability. Similarly, the reference method showed high reproducibility between test sessions, although a small but statistically significant difference between measurements was observed. Overall, both methods demonstrated strong and clinically acceptable measurement stability.

#### 3.1.2. Stereovision

Test–retest reliability of stereopsis assessments was evaluated in 24 participants using binary pass–fail classification. The test–retest subgroup predominantly consisted of participants with preserved stereopsis. All stereopsis tests demonstrated high reproducibility between sessions, with only a small number of classification changes observed. Agreement metrics indicated excellent consistency for both the reference and index tests; however, the wide confidence intervals of Cohen’s kappa statistics are likely related to the moderate sample size. Detailed reliability outcomes are presented in Table 3B.

**Table 3:** Test–retest reliability outcomes for visual acuity and stereopsis measurements. (**A**) Visual acuity reliability for pooled, non-truncated monocular and binocular VA data. (**B**) Stereopsis reliability (binary pass–fail classification).

(**A**)						
**VA Test**	**Bias** **(95% CI)**	**SD** **(Bias)**	**Limits of Agreement** **(Lower; Upper, 95% CI)**	**Mean (SD) for** **Test and Retest,** **Paired Sample *t*-Test:** ***p*** **-Value, *t*-Stat**	**ICC** **(95% CI)**	**ICC,** ***p*** **-Value**
Reference method	0.048 (0.030–0.065)	0.075	−0.10 (−0.13–−0.068); 0.19 (0.16–0.23)	−0.029 (±0.21); −0.076 (±0.20) <0.001 (5.37)	0.90 (0.85–0.94)	<0.001
Index method	0.022 (−0.0036–0.047)	0.107	−0.19 (−0.23–−0.144); 0.23 (0.19–0.28)	−0.14 (±0.18); −0.16 (±0.15) 0.092	0.79 (0.68–0.86)	<0.001
(**B**)						
**Test**	**Session 1** **Summary**		**Session 2 Summary**	**Identical Classification *n* (%)**	**Cohen’s κ (95% CI)**	
Reference method (TNO stereotest)	95″ range: 60–480″; 95% CI: 53.46–136.54		82.5″ range: 60–240″ 95% CI: 55.76–109.24	24/24 (100%)	1	
Index method SRDS	9.7/10 stimuli		9.4/10 stimuli	24/24 (100%)	1	
Index methodDRDS	9.0/10 stimuli		8.8/10 stimuli	23/24 (95.9%)	0.647 (0.013 1.00)	

Reference method: ETDRS-style Landolt C chart. Index method: tablet-based application. Bias represents the mean difference between test and retest measurements. Limits of agreement were calculated according to the Bland–Altman methodology. ICC: intraclass correlation coefficient. Stereopsis outcomes were analyzed using binary pass–fail classification. Identical classification indicates participants receiving the same classification across sessions. Agreement beyond chance was quantified using Cohen’s kappa.

### 3.2. Clinical Cross-Sectional Group

#### 3.2.1. Visual Acuity

Altogether 63 patients completed all VA tests under both monocular and binocular viewing conditions, resulting in 189 VA data points per method. The Q-Q plot comparing the observed z-scores to the expected normal distribution suggests that the reference methods’ monocular VA data approximated a normal distribution, with only minor deviations at the tails. 

**Figure 1:**
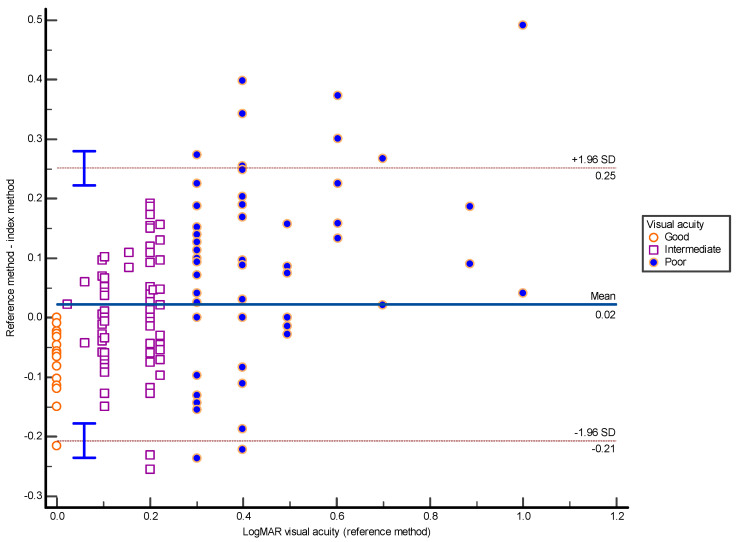
Bland–Altman plot showing agreement between reference and index VA methods. The x-axis displays logMAR VA from the reference method; the y-axis shows the difference between the two methods (reference-index). Each data point represents one of three measurements per participant: binocular, right eye, or left eye. Solid blue line: mean difference (bias); red dashed lines: 95% limits of agreement (LoA, mean ±1.96 SD); vertical blue bars: confidence intervals. Orange circles: good VA (logMAR = 0); purple squares: intermediate VA (0.0 < logMAR < 0.3); blue dots: poor VA (logMAR ≥ 0.3). VA: visual acuity.

Since the reference method did not measure into the hyperacuity range (better-than-normal vision), values equal to or better than 0 logMAR (*n* = 50) were truncated at 0 for both methods. Monocular results differed slightly between methods (0.20 vs. 0.23 logMAR, *p* = 0.0031), and binocular results were nearly identical (0.10 vs. 0.097 logMAR, *p* = 0.80). To evaluate agreement, scores from both tests were compared with the Bland–Altman method, and VA data from all three viewing conditions (binocular, right, and left eye) were pooled into a single dataset (presented in Figure 1 and Table 4). The overall mean difference between the two methods was 0.022 logMAR, indicating a small and clinically acceptable average bias.

When grouped by visual performance, the poor VA subgroup (logMAR ≥ 0.3) showed a larger mean difference (0.092 logMAR), whereas results in the intermediate VA group (0 < reference < 0.3 logMAR) were nearly identical (−0.0061 logMAR). A paired *t*-test confirmed that the difference in the poor VA subgroup was statistically significant (*p* < 0.01), while no significant difference was observed in the intermediate VA range.

In addition, proportional bias was formally assessed by linear regression of the Bland–Altman differences (index-reference) on the between-method means. This analysis showed no evidence of fixed bias, as the intercept did not differ from zero (intercept = −0.0002, 95% CI −0.023 to 0.023, *p* = 0.984). However, a statistically significant proportional bias was observed (slope = −0.125, 95% CI −0.216 to −0.035, *p* = 0.0068), indicating that the magnitude of the between-method difference varied with the level of visual acuity.

#### 3.2.2. Stereovision

**Figure 2:**
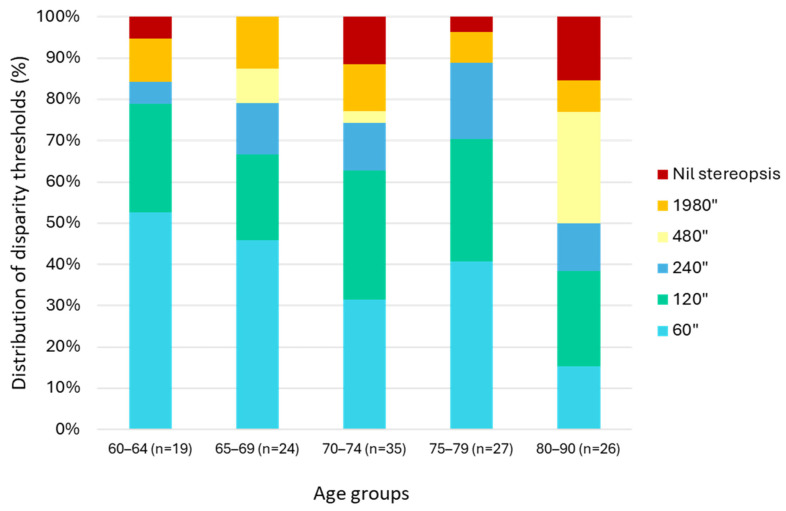
Distribution of TNO stereopsis thresholds across age groups (*n* = 131). Bars represent the relative proportion (%) of participants in each threshold category (60″–1800″ and nil stereopsis). There was no progressive increase in higher thresholds with advancing age (χ^2^ = 31.062, df = 20, *p* = 0.0544), showing only marginal significance.

Stereopsis thresholds measured by the TNO test showed considerable inter-individual variability across the examined age range (60+ years) as presented in Figure 2. However, no statistically significant trend was observed between age groups (χ^2^ = 31.062, df = 20, *p* = 0.0544), indicating that within this older adult cohort, stereoacuity did not decline progressively with advancing age.

The mean stereoacuity threshold among participants with measurable stereopsis was 342.15″ (95% CI: 237.33–446.97″), while 10 individuals demonstrated no stereopsis. Although thresholds ranged widely (0–1800″), this variability was not systematically associated with age.

Agreement analyses across multiple TNO disparity cutoffs (see Appendix A) demonstrated that the 480″ level provided the highest correspondence with index test outcomes. Given that this value also aligns with the upper range of stereoacuity typically reported in older populations [26,27,28], the 480″ threshold was selected as the primary reference criterion for dichotomous classification.

**Figure 3:**
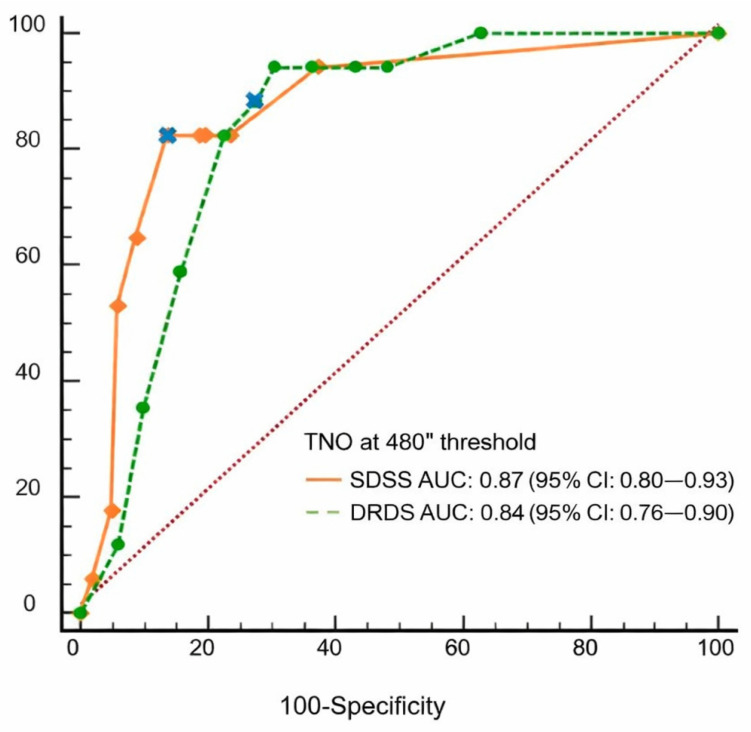
ROC analysis of the index stereotests (SRDS and DRDS). Participants were categorized into impaired and intact stereovision groups based on TNO results. Orange rhombuses represent SRDS criterion values, green dots represent DRDS criterion values, and blue crosses indicate the selected screening cutoff (5/10 correct responses for both SRDS and DRDS) optimized for high sensitivity. Using this cutoff, SRDS yielded a sensitivity of 82.4% (95% CI: 56.6–96.2) and specificity of 81.4% (95% CI: 70.0–89.3), whereas DRDS yielded a sensitivity of 84.0% (95% CI: 63.9–95.5) and specificity of 72.3% (95% CI: 62.2–81.1). The 480″ TNO criterion yielded the highest AUC values, supporting its use as the reference threshold (see also Appendix A, Table A1). The diagonal dotted line represents the chance-level discrimination.

Diagnostic performance of SRDS and DRDS for detecting impaired stereovision defined by the selected TNO threshold was evaluated using ROC analysis. Both index tests demonstrated strong discriminatory performance relative to the TNO classification using the predefined screening cutoff. Detailed ROC characteristics, including sensitivity and specificity values, are presented in Figure 3. 

Paired comparison analyses showed that both index stereotests identified a greater proportion of participants with impaired stereovision compared with the TNO test at the selected reference threshold (Appendix A Table A1). These differences were statistically significant based on McNemar’s test (SRDS: *p* = 0.0009; DRDS: *p* < 0.0001). Comparison of overall discriminatory performance between SRDS and DRDS using DeLong’s test demonstrated no statistically significant difference between ROC curves (AUC difference = 0.033, SE = 0.039, 95% CI: −0.042 to 0.109, *p* = 0.39).

## 4. Discussion

This study assessed visual acuity (VA) and stereovision (SV) in adults over 60 years of age using a tablet-based system as index methods and compared their performance with established clinical reference tests, namely the ETDRS-style Landolt C chart for VA and the TNO stereotest for SV.

Test–retest analysis demonstrated good reliability for VA measured with both methods. The index method showed no statistically significant difference between repetitions (*p* = 0.092) and a good repeatability with an ICC of 0.79. The findings are consistent with previous research that reports the robustness of computerized single-optotype VA tests [29,30].

Agreement between the index and reference VA methods was clinically acceptable across the full dataset, with mean bias remaining within ±0.1 logMAR for all acuity ranges, meeting the clinically acceptable threshold [31,32]. In the intermediate VA range of the clinical cross-sectional group, agreement was even more stringent and met the 0.05 logMAR criterion specified for identical optotypes in ISO/TR 19498 [33]. Despite this overall agreement, Bland–Altman regression revealed proportional bias, indicating that between-method differences increased as visual acuity worsened. Specifically, at lower VA levels, the index method tended to yield systematically better (i.e., lower logMAR) values than the ETDRS reference.

The index method uses single optotype presentation combined with an adaptive threshold-search algorithm (Best PEST), whereas the ETDRS chart relies on line-based presentation with fixed size steps and stricter stopping criteria. Single-optotype approaches are widely used in digital screening tools, and provide reliable VA estimates while enabling rapid assessment [25,29,34,35]. Although line-based presentation may theoretically induce crowding effects, this has minimal impact on adult foveal VA under standard ETDRS spacing conditions [30,36,37].

In addition, the Best PEST adaptive algorithm uses 59 optotype sizes for semi-continuous estimation enhancing sensitivity in lower VA ranges and sampling the steepest segment of the psychometric function [22,25,34]. In contrast, ETDRS relies on 18 discrete size steps [9], and a fixed criteria may result in floor effects. Together, these methodological differences plausibly explain the observed proportional bias without implying true differences in visual performance

Although meaningful differences exist in the lower range (≥0.3 logMAR), appropriate correction factors may reduce these discrepancies, therefore, validation in clinical populations spanning a broad range of VA is warranted. Taken together, the observed bias of VA is more likely attributable to methodological variations—such as test format and delivery (e.g., adaptive versus fixed staircase, tablet versus printed chart)—rather than genuine differences in visual function the measure.

With respect to stereovision, the index method differs conceptually from the disparity threshold-based TNO test. The static and dynamic random-dot stereogram (SRDS) functions as a pass/fail screening tool at a fixed disparity level rather than providing continuous stereoacuity threshold, enabling rapid assessment of gross stereopsis. This design facilitates large-scale or remote screening and complements threshold-based methods like TNO.

Our findings, together with previous reports, indicate that stereovision declines gradually with age but reaches a plateau beyond 60 years, suggesting relatively stability in this population [24,25,26,27,28]. In the present cohort, the mean TNO threshold was 342.15″ (95% CI: 237.33–446.97), and the optimal cutoff of 480″ closely matched published median values of 408″ and 480″ for the 61–70 and 71–80-year groups, respectively. This convergence supports the applicability of a 480″ TNO threshold for distinguishing normal from impaired stereopsis in older adults.

Both the index and reference stereotests demonstrated high test-retest reproducibility, with binary agreement exceeding 95%, underscoring their reliability in elderly participants. The high proportion of “pass” outcomes, however, suggest a possible ceiling effect in this generally healthy cohort.

In the clinical cross-sectional group, ROC analysis supported the clinical relevance of the 480″ cutoff, yielding good diagnostic accuracy for both static and dynamic index tests. While the index methods identified more cases of impaired stereovision than the TNO, particularly for dynamic stimuli, this likely reflects higher sensitivity rather than reduced specificity. These findings are consistent with previous studies demonstrating the ability of this screening approach to detect clinically relevant stereovision deficits [13,20,21].

Given the established association between reduced stereovision, falls and fracture risk in older adults [1,8,12,13], accurate and reproducible screening tools are essential for early identification. 

If confirmed in future studies, referral for comprehensive ophthalmologic examination may be considered in individuals with reduced visual acuity (≥0.3 logMAR) or impaired stereovision according to the applied screening criteria, while periodic reassessment may be appropriate in the absence of abnormal findings [38,39,40]. This proposed pathway, however, requires prospective validation [8,12,40,41,42,43].

### Limitations

First, in accordance with common clinical practice, ETDRS-LC-measurements were terminated at 0.0 logMAR, and for consistency, Best-PEST VA estimation results in the clinical cross-sectional group were similarly truncated for data analysis. Therefore, comparison at 0 and lower logMAR values was statistically not feasible. 

Second, due to logistical constraints, retest measurements could not be performed on the clinical cross-sectional group; instead, an additional group of 24 low-risk older adults (aged 60+) was assessed, with full-range VA measurements. Therefore, data from the two groups are not directly comparable. 

Third, because of differing threshold definitions (stereoacuity threshold vs. number of correctly identified static and dynamic RDSs), results from the two stereotests included in the study are not directly comparable. 

Fourth, pooling measurements from both eyes and binocular testing may introduce inter-eye correlation. Although this may limit independence of observations, the approach was applied consistently across methods and was considered appropriate for measurement agreement analysis. 

A further limitation of the present study is the relatively small sample size of the test–retest group. This measurement was not designed to support formal categorical agreement testing; consequently, the number of discordant cases in SV testing was low, particularly due to the generally good visual performance of the healthy older participants. Future studies including a larger and more heterogeneous sample are needed to allow robust categorical agreement analyses for the stereovision tests. 

Furthermore, the study sample was drawn from ambulatory, cognitively intact older adults using convenience sampling, which limits generalizability to institutionalized or cognitively impaired populations. Future studies should extend evaluation to higher-risk groups including post-fall and long-term care elderly populations.

## 5. Conclusions

In summary, the tablet-based index method demonstrated clinically acceptable agreement with established reference standards for the assessment of visual acuity and stereovision, and proved applicable across different care settings. Although some differences were observed at lower visual acuity levels, test performance was consistent within the range typically relevant for screening purposes.

The findings support the feasibility of using the tablet-based method for the early identification of visual impairment in older adults. With further validation in broader and higher-risk populations, such tools may become valuable components of clinical and community-based screening initiatives.

From an implementation perspective, the tablet-based assessment should be interpreted as a screening and triage instrument rather than a stand-alone determinant of fall risk. Pending confirmation in prospective studies, referral for comprehensive eye examination may be considered even in asymptomatic individuals when visual acuity falls below commonly applied thresholds for impaired vision in older adults or when stereopsis is reduced. In the absence of abnormal findings, periodic reassessment may be sufficient. This proposed screening pathway requires further prospective validation.

## Figures and Tables

**Table 1:** Baseline characteristics of participants included in the data analysis. VA and SV as measured by reference methods.

	Number of Patients	Mean Age (yrs)	Female (%)	Mean VA (logMAR)	Stereoacuity (arcmin)	Inclusion Criteria	Exclusion Criteria
Test–retest group	24	65.96 SD = ±5.94	45.8	−0.052(SD: ±0.20)	95″ (95% CI: 53.46–136.54)	- age ≥ 60 years - monocular VA on any eye for group 1: ≤0.85 logMAR for group 2: ≤1.3 logMAR - providing informed consent	- untreated severe eye disease - eye surgery within the past three months - cognitive impairment withdrawal
Clinical cross-sectional group	135	72.73 SD = ±6.95	70	0.19 (SD: ±0.20)	342.15″ (95% CI: 237.33–446.97)		

**Table 2:** Summary of VA and SV test characteristics including the number of participants evaluated.

Test Name	Stimuli	Channel Separation	Type of Test	Manufacturer	Dot Size, Viewing Distance (meters)	Luminance of Background (cd/m^2^)	Possible Results	Cutoff	Nr. of Participants (Test–Retest and Clinical Cross-Sectional Groups)
	Visual acuity testing								
Index VA method	Landolt C	N/A	high contrast optotype	EuvisionTab^®^ v0.32, Euvision Ltd., Pécs, Hungary	N/A, 5.0	165	1.3 to −0.44 logMAR	Best PEST: steepest part of the psychometric function	24 and 63
Reference VA method (ETDRS-style)	Landolt C	N/A	high contrast optotype	Visionix, VX 22-LP Chart display, Visionix Ltd., Jerusalem, Israel	N/A, 5.0	207	1.3 to −0.4 logMAR	3 /5 correct	24 and 63
	Stereovision testing								
Index SV method	Snellen E presented in SRDS and DRDS	anaglyphic	global, random dot	EuvisionTab^®^ v0.32, Euvision Ltd., Pécs, Hungary	420″ 0.25–0.30	N/A	0–10 out of 10 presentations	5/10 5/10 correct	24 and 119
Reference SV method	Plates V and VI, “pancake”	anaglyphic	global, random dot	Lameris Ootech B.V., Utrecht, The Netherlands	108″ 0.40	N/A	Nil stereopsis, 1800″ 480″ 240″ 120″ 60″	480″	24 and 131

VA: visual acuity; SV: stereovision; ″: arcsecond; Best PEST: Best Parameter Estimation by Sequential Testing; ETDRS: Early Treatment Diabetic Retinopathy Study; SRDS: static random dot stereogram; DRDS: dynamic random dot stereogram; N/A: not applicable.

**Table 4:** Agreement between index and reference VA tests, based on Bland–Altman analysis and paired-sample *t*-tests.

VA Range (logMAR)	N. of Tests	Bias (95% CI)	Lower LoA (95% CI)	Upper LoA (95% CI)	Mean (SD) Reference and Index Test, Paired *t*-Test
All ranges	189	0.022 (0.0057 to 0.039)	−0.206 (−0.24 to −0.18)	0.25 (0.22 to 0.28)	0.19 (0.20); 0.17 (0.18) *p* = 0.0088
Poor: reference ≥ 0.3	55	0.092 (0.049 to 0.14)	−0.22 (−0.29 to 0.15)	0.41 (0.33 to 0.48)	0.44 (0.18); 0.35 (0.20) *p* = 0.0001
Intermediate: 0 < reference < 0.3	75	−0.0061 (−0.019 to 0.0072)	−0.16 (−0.18 to 0.14)	0.15 (0.12 to 0.17)	0.15 (0.056); 0.14 (0.1021) *p* = 0.44
Good VA reference = 0	59	0.047 (0.017 to 0.076)	−0.17 (−0.23 to −0.12)	0.27 (0.22 to 0.32)	0.00 (0.0); 0.024 (0.049) *p* = 0.0003

Presented are the number of measurements (N), mean bias (with 95% CI), 95% limits of agreement (LoA), and mean values (SD) across all measurements and the three VA subgroups.

## Data Availability

The datasets used and analysed during the current study are available from the corresponding author on reasonable request.

## Abbreviations

The following abbreviations are used in this manuscript:

″	Second of Arc, Arcsecond, Arcsec
AUC	Area Under the Curve
Best PEST	Best Parameter Estimation by Sequential Testing
DRDS	Dynamic Random Dot Stereogram
ETDRS	Early Treatment Diabetic Retinopathy Study
LoA	Limits of Agreement
NA	Not Applicable
OR	Odds Ratio
ROC	Receiver Operating Characteristic
RDS	Random Dot Stereogram
SRDS	Static Random Dot Stereogram
SV	Stereovision
VA	Visual Acuity

## Appendix A

### Determination of the Optimal TNO Reference Threshold

Because no age-specific consensus disparity threshold has been established for elderly individuals using the TNO stereotest, and previous reports only suggest an approximate stereoacuity range of 240″–480″ in this population [26,27,28,29], an empirical evaluation of alternative reference cutoffs was performed with the data of this study.

To address this methodological uncertainty, we performed receiver operating characteristic (ROC) analyses across all available TNO disparity levels (60″, 120″, 240″, 480″, and 1980″) using both SRDS and DRDS outcomes as index classifications. For each potential TNO cutoff, pass–fail agreement with the predefined index criterion (≥5/10 correct responses) was evaluated, and the corresponding area under the curve (AUC) values were calculated.

Among the examined disparity thresholds, the 480″ level yielded the highest AUC values for both SRDS and DRDS, indicating the strongest discriminatory performance and agreement between the reference and index methods (see Appendix A Table A1). Lower thresholds resulted in reduced sensitivity, whereas higher thresholds decreased specificity.

These findings support the selection of 480″ as the most appropriate reference criterion within this older adult cohort. Although standardized TNO cutoffs for elderly populations are lacking, the present data provide empirical justification for this threshold and reinforce its consistency with the upper stereoacuity range reported in prior studies [26,27,28,29].

**Table A1:** Comparison of stereovision measured by TNO, SRDS, and DRDS.

TNO Threshold		TNO	SRDS (Pass/Fail)	DRDS (Pass/Fail)
60′	Pass	63	54/9	46/17
	Fail	56	31/25	26/30
	AUC (95%CI)	-	0.70 (0.61–0.78)	0.68 (0.59–0.76)
120′	Pass	78	69/9	59/19
	Fail	41	16/25	13/28
	AUC (95%CI)	-	0.78 (0.69–0.85)	0.74 (0.66–0.82)
240′	Pass	94	78/16	68/26
	Fail	25	7/18	4/21
	AUC (95%CI)	-	0.80 (0.72–0.87)	0.82 (0.74–0.88)
480′	Pass	102	82/20	71/31
	Fail	17	3/14	1/16
	AUC (95%CI)	-	0.87 (0.80–0.93)	0.84 (0.76–0.90)
1980′	Pass	112	82/30	71/41
	Fail	7	3/4	1/6
	AUC (95%CI)	-	0.75 (0.67–0.83)	0.79 (0.70–0.86)
NIL stereopsis TNO		7	3/4	1/6
NIL stereopsis SRDS		29/4	33	33
NIL stereopsis DRDS		41/6	14/33	47

Pass-fail data and area under the curve data are presented at different cutoff values of TNO, the stereotest (TNO threshold). The best match between the TNO and RDS tests is indicated in italics. NIL stereopsis: absence of measurable stereopsis; SRDS: static random dot stereogram; DRDS: dynamic random dot stereogram; AUC: area under the curve.
